# Skull Base Osteomyelitis: A Single-Center Experience

**DOI:** 10.7759/cureus.20162

**Published:** 2021-12-04

**Authors:** Furqana Akhtar, Jhanzeb Iftikhar, Musa Azhar, Aun Raza, Faisal Sultan

**Affiliations:** 1 Infectious Diseases, Bahria International Hospital, Lahore, PAK; 2 Medical Oncology, Shaukat Khanum Memorial Cancer Hospital and Research Centre, Lahore, PAK; 3 Infectious Diseases, Shaukat Khanum Memorial Cancer Hospital and Research Centre, Lahore, PAK

**Keywords:** sbo, craniofacial osteomyelitis, invasive sinusitis with intracranial extension, malignant otitis externa, skull base ostemyelitis

## Abstract

Background

Skull base osteomyelitis (SBO) is an uncommon entity and carries a high mortality rate. It can be be odontogenic, sinogenic, or otogenic in origin, in addition to being a complication of skull surgery/trauma. *Pseudomonas* is one of the most commonly identified pathogen. The goal of the study is to describe the clinical spectrum, microbiologic characteristics, treatment, and its response among different patients with SBO. In addition, we compared the outcomes of bacterial and fungal osteomyelitis.

Methodology

This is a single-center retrospective analysis of patients with SBO who presented to Shaukat Khanum Memorial Cancer Hospital & Research Centre Lahore, Pakistan between January 1998 and September 2019. A total of 15 patients with SBO were identified.

Results

SBO was common in males (79.9%) with a high body mass index. Diabetes mellitus was the most common co-morbid condition (46.62%). Bacterial etiology was seen in 46.62% and fungal isolate was detected in 6.66% of the patients; 26.64% were culture-negative and the remaining had a mixed culture. The mean duration of treatment was 17.58 ± 10.85 weeks. Overall, five (33.3%) patients were cured and did not have a recurrence of symptoms at six months, while three (19.98%) had a recurrence of symptoms at six months from the end of the treatment; six (39.96%) patients were lost to follow-up.

Conclusions

Patients with SBO can present with various conditions, and early identification of the condition and a positive culture growth can guide optimal treatment.

## Introduction

Cranial osteomyelitis or skull base osteomyelitis (SBO) is an uncommon entity with high mortality (10-20%). Chronic or inadequately treated infection of the craniofacial skeleton is usually the etiology. Osteomyelitis can be odontogenic, sinogenic, or otogenic in origin; however, it can also be a complication of direct injuries to the skull (trauma/surgery) [1]. Several types of SBO have been described in the literature such as central, atypical clival, or pediatric SBO [2]. Advanced age, male gender, and diabetes are a few of the well-established risk factors for developing SBO [3].

*Pseudomonas aeruginosa* is one of the most commonly encountered bacterial pathogens. Others include methicillin-resistant *Staphylococcus aureus* (MRSA)/methicillin-susceptible *Staphylococcus aureus* (MSSA), *Streptococci*, and *Enterobacteriaceae*. *Aspergillus*, *Scedosprium*, and *Zygomycetes* are the most common fungal isolates. Proximity to the brain, complex craniofacial anatomy, and cosmetic concerns render SBO a therapeutic challenge [4,5].

Due to the lack of large, adequately powered epidemiological studies, deficient established guidelines for the treatment, and high mortality, SBO is a diagnostic and therapeutic dilemma and warrants further investigation. In this study, we aim to describe the clinical spectrum, microbiologic characteristics, treatment, and response among patients with SBO. In addition, we compare the outcomes of bacterial versus fungal osteomyelitis.

## Materials and methods

Study design and data collection

This is a retrospective descriptive study. A 20-year (January 2000 to September 2020) retrospective review of patients with SBO presenting to Shaukat Khanum Memorial Cancer Hospital and Research Centre, Lahore was performed. Patient charts were searched using keywords “skull base osteomyelitis,” “malignant otitis externa,” “invasive sinusitis with the intracranial extension,” and “craniofacial osteomyelitis” from the electronic database. Demographic, clinical, laboratory, radiology, the treatment offered, and response to treatment data were reviewed. Outcomes were categorized as disease-free survival (cured), recurrence, and mortality (all-cause) at six months.

Inclusion and exclusion criteria

All adult patients (aged 18 and above) presenting with a diagnosis of SBO (clinically or radiologically established) were included in the study. Patients aged less than 18 years and with no involvement of the skull bone were excluded.

Statistical analysis

Statistical analysis was carried out using the Statistical Package for the Social Sciences (SPSS) software, version 20.0 (IBM Corp., Armonk, NY, USA).

## Results

A total of 15 patients were identified to have SBO. Of the 15 patients, 12 (79.9%) were males with a mean age of 58.13 ± 14.3 years and a mean body mass index (BMI) of 26.0 ± 4.3 kg/m^2^. Diabetes mellitus was the most common co-morbid condition. Moreover, seven (46.62%) patients had diabetes with complications or end-organ damage. However, hemoglobin A1c (HbA1C) was not recorded in all patients. The mean HbA1C was 6.9% (range: 6.1-8.8%) among the available data. Other comorbid conditions are listed in Table 1.

**Table 1:** Baseline characteristics and laboratory parameters, N (%). HbA1C: hemoglobin A1c; PAS: periodic acid Schiff; GMS: Gomori methenamine silver

	N (%)
Male	12 (79.9%)
Body mass index (kg/m^2^)	26.0 ± 4.3
Age (years)	58.13 ± 14.3
Co-morbid conditions	
Diabetes mellitus*	10 (66.6%)
Hypertension	1 (6.6%)
Depression	1 (6.6%)
Malignancy	1 (6.66%)
Multiple	8 (53.28%)
None	1 (6.66%)
Charlson Comorbidity Index (Estimated 10-year survival)	
0 (98%)	1 (6.66%)
1 (96%)	2 (13.32%)
2 (90%)	1 (6.66%)
3 (77%)	1 (6.66%)
4 (53%)	2 (13.32%)
5 (21%)	3 (19.98%)
6 (2%)	3 (19.98%)
7 (0%)	1 (6.66%)
9 (0%)	1 (6.66%)
Laboratory parameters	
HbA1C	6.90%
White blood cell count	8.1 ± 2.64
C-reactive protein	42.8 ± 40
Erythrocyte sedimentation rate	70.75 ± 14.38
Histopathology	
Acute and chronic inflammation, negative for PAS and GMS	6 (39.96%)
Chronic granulomatous inflammation with septate hyphae	2 (13.32%)
Chronic inflammation with aseptate hyphae	3 (19.98%)
Diabetes mellitus alone and with other co-morbidities	1 (6.66%)

SBO as a contiguous infection from the ears was observed in eight (53.28%) patients, of which two (13.32%) patients had otitis media/mastoiditis, three (19.98%) had malignant otitis externa, and three (19.98%) had involvement of the middle ear as well as the external ear. Overall, three (19.98%) patients had paranasal sinuses as the primary site of infections, that is, one each for trauma, post-surgery, orbital cellulitis, and nasopharyngeal mass.

SBO patients presented with a wide range of symptoms. The mean duration of symptoms before presentation was 11 ± 15.36 months. Majority of the presenting symptoms included headache [10 (66.6%)], nausea [eight (53.28%)], otalgia/otorrhea [eight (53.28%)], and Bell’s Palsy [seven (46.62%)], as shown in Table 2.

**Table 2:** Symptoms at presentation.

	N (%)
Headache	10 (66.6%)
Nausea/anorexia	8 (53.28%)
Otalgia, otorrhea, and hearing loss	8 (53.28%)
Bell’s palsy	7 (46.62%)
Facial swelling and pain	4 (26.64%)
Cranial nerve palsies other than Bell’s palsy	2 (13.32%)
Multiple cranial nerve palsies	2 (13.32%)
Ataxia	2 (13.32%)
Limb weakness	0
Diplopia	2 (13.32%)
Eye pain, swelling, and visual loss	2 (13.32%)
Peri-orbital swelling and proptosis	1 (6.66%)
Blocked nose, epistaxis, and rhinorrhoea	3 (19.98%)

Radiological findings and skull involvement

Imaging was the most important aspect of investigations. In our study, three (19.98%) patients underwent computed tomography (CT) scan, four (26.64%) underwent MRI, eight (53.28%) required both, and one (66%) underwent positron emission tomography (PET) scan. Radiological findings mainly included erosive/destructive lesions in the area involved, mastoid opacification with air-fluid levels, and post-contrast enhancement. In one case, there was a large destructive lesion in the nasopharyngeal area with extensive involvement of multiple bones of the base of the skull. The biopsy was inconclusive twice. A PET scan was performed which showed a hypermetabolic mass at the base of the skull with extension along the styloid process, suggestive of fungal infection. Only one patient had evidence of thrombosis of the cavernous sinus. Temporal bone was the most frequently involved bone in nine (59.94%) patients. Three (19.98%) patients had involvement of multiple bones, namely, the petrous part of the temporal bone along with the occipital bone, the frontoparietal and temporal bone, and the greater wing of the sphenoid, temporal, and frontal bone (orbital and peripheral nervous system involvement).

Microbiological data

Isolation of the causative microbial agents is crucial in the management of any disease with an infectious etiology. Out of 15 patients, 11 (73.26%) had positive cultures, with bacterial etiology in seven (46.62%) and fungal in one (6.66%) case (*Rhizopus* spp.) However, three (19.98%) patients had both bacterial as well as fungal etiologies. In four (26.64%) cases of culture-negative SBO, histology was suggestive of fungus (*Aspergillus*) in three cases, and one patient received both antibacterial and antifungal treatment with subsequent improvement.

*P. aeruginosa* was identified as the causative agent in three (19.98%) cases, MRSA in two cases, and MSSA in one case. Four (26.64%) patients had polymicrobial growth involving MRSA and *P. aeruginosa*; *P. aeruginosa* and *Streptococcus* spp.; *P. aeruginosa* and *Enterococcus*; and *Proteus*, *P. aeruginosa*, and MRSA.

The diversity in the sensitivity profile of *P. aeruginosa* and MRSA is shown in Tables 3, 4, respectively. Eight (53.28%) patients were treated for fungal SBO, one patient for *Rhizopus* (histology as well as culture both positive), and seven (46.62%) for *Aspergillus*; culture-proven in three (19.98%) cases and histology with branching septate hyphae in four (26.64%) cases.

**Table 3:** Sensitivity profile of Pseudomonas aeruginosa (N = 11).

	N (%)
Ciprofloxacin	5 (45.45%)
Ceftazidime	6 (54.54%)
Cefepime	4 (36.36%)
Piptazobactam	6 (54.54%) sensitive, 2 (18.18%) intermediate
Meropenem	6 (54.54%)
Imipenem	7 (63.63%)
Amikacin	5 (45.45%)
Colistin	11 (100%)

**Table 4:** Sensitivity profile of MRSA (N = 6). MRSA: methicillin-resistant *Staphylococcus aureus*

	N (%)
Doxycycline	3(50%)
Cotrimoxazole	4 (66.66%)
Clindamycin	2 (33.33%)
Teicoplanin	6 (100%)
Vancomycin	6 (100%)

Treatment and outcome

The mean duration of treatment was 17.58 ± 10.85 weeks. Five (33.3%) patients were cured and did not have recurrence of symptoms at six months, while three (19.98%) had a recurrence of symptoms at six months from the end of treatment. Six (39.96%) patients were lost to follow-up. One patient died and the likely cause of death was advanced central nervous system involvement and lack of source control (Table 5). Antifungal treatment duration was 21.47 weeks versus 11.72 weeks in cases of confirmed or presumed bacterial infections.

**Table 5:** Treatment, duration, and outcome. SBO: skull base osteomyelitis; HP: histopathology; *P. aeruginosa*: *Pseudomonas aeruginosa*; MRSA: methicillin-resistant *Staphylococcus aureus*; MSSA: methicillin-sensitive *Staphylococcus aureus*

No.	Pathogen	Treatment offered	Duration	Total duration	Outcome
1	Culture-negative SBO (HP: *Aspergillus*)	Voriconazole	24 weeks	24+ weeks	Cured
2	P. aeruginosa	Pipercillin/tazobactam	4 weeks	4 weeks	Cured
3	MRSA	Linezolid and co-trimoxazole	10 + 9 weeks	19 weeks	Cured
	Aspergillus	Voriconazole	19 weeks	19 weeks	
4	P. aeruginosa	Pipercillin/tazobactam + imipenem	18 + 3 weeks	21 weeks	Death
5	MRSA	Vancomycin + linezolid + doxycycline	6 + 3 + 4 weeks	13 weeks	Recurrence
	P. aeruginosa	Ciprofloxacin + meropenem	6 + 1 weeks	7 weeks	
6	MSSA	Co-amoxiclav + cephalexin + cephazoline	21 weeks	21 weeks	Recurrence
7	*P. aeruginosa* and *Streptococcus* spp.	Pipercillin/tazobactam + meropenem	10 + 8 weeks	18 weeks	Recurrence
8	Rhizopus arrhizus	Amphotericin B + posaconazole	3 + 12 weeks	15 weeks	Cured
9	P. aeruginosa	Pipercillin/tazobactam + imipenem + ciprofloxacin	1 + 8 weeks	9 weeks	Cured
10	Culture-negative SBO (HP: *Aspergillus*)	Voriconazole	32.5 weeks	32.5 weeks	Cured
11	P. aeruginosa	Pipercillin/tazobactam	4 weeks	4 weeks	Death
	Enterococcus				
12	Culture-negative SBO (HP: *Aspergillus*)	Voriconazole	32 weeks	32 weeks	Cured
13	MRSA	Vancomycin + linezolid + cotrimoxazole	6 weeks	6 weeks	Cured
	Aspergillus	Amphotericin B + posaconazole	4 + 32 weeks	36 weeks	
14	Culture-negative SBO	Ertapenem	6 weeks	6 weeks	Recurrence
		AmphotericinB + voriconazole	2.3 + 8 weeks	10.3 weeks	
15	*Proteus*, *Pseudomonas*, and MRSA	Piptazobactam + imipenem + linezolid + clindamycin	2 + 2 + 2 + 2 weeks.	8 weeks	Recurrence

## Discussion

SBO is an important entity owing to the complexity of infection, proximity to brain tissue, and aesthetic concerns. Due to the rarity of the disease, it is a challenge for physicians to recognize the clinical features of SBO and maintain a high index of suspicion toward the condition. However, treatment is often difficult as there are no well-established clinical guidelines along with a lack of good quality clinical data and an increased fatality rate [1].

Khan et al. reported a 1.7:1 male-to-female ratio with an average age of 15-70 years [2]. However, in our study, marked male predominance was noted with a male-to-female ratio of 4:1 and a mean age of 58.13 ± 14.3 years. Diabetes mellitus was an important risk factor, especially when poorly controlled with end-organ damage. Most patients (13/15, 86.66%) developed the disease as a spread of an infection from the contiguous site, mainly as a complication of otogenic infection followed by sinusitis. However, it resulted from trauma and postcranial surgery in two (14.34%) patients, according to existing data [1-3]. One large study from India reported headache as the most common manifestation, followed by otalgia and otorrhea, and a blocked nose and nasal discharge. Bell’s palsy was the most common residual neurological damage, and its occurrence was slightly higher in our cohort than the existing data (56% v3s. 40%) [1,4]. Patients with multiple cranial nerve palsies had worse outcomes as the surgical intervention was not possible due to proximity to the brain and extensive involvement, which is also well documented in the literature [6].

In diagnostic imaging, MRI with contrast is the most important tool for diagnosing craniofacial osteomyelitis. Key findings include a combination of lateral extension, increased T2 signal, lack of architectural distortion, and enhancement greater than or equal to mucosa [7]. Overall, 12 MRI results of our patients had the above findings in different combinations, destructive lesions with extension into dura mater in two patients and involvement of brain parenchyma in one, which confers poor prognosis [8]. One of our patients had diagnostic uncertainty, for which a PET scan was performed. FDG PET-CT can be a sensitive tool in analyzing patients with SBO and can play a crucial role in evaluating treatment response [9].

In our case series, there was only one patient where no pathogen was isolated. In almost half of the patients, *P. aeruginosa* is isolated. *S. aureus* was the second most common, followed by *Proteus*, *Enterococcus*, and *Klebsiella* species, which is consistent with earlier data from different studies [3-4,10,11]. Increasing resistance of isolates and polymicrobial growths are associated with worse clinical outcomes [1,6,12]. Fungal infections were more common in patients who developed SBO as a contiguous infection from the nose and paranasal sinuses. *Aspergillus* species were the most common fungal isolate, as described by Blyth and colleagues [4]. Recurrence and death were more common in cases with a bacterial etiology, especially when polymicrobial pathogens were isolated in cultures. The sensitivity profile of bacteria differs in different studies and mainly depends upon local epidemiology, antimicrobial resistance pattern, and broad-spectrum antimicrobials. Regarding the sensitivity profile of microbes isolated, all *P. aeruginosa* isolates were extended-spectrum beta-lactamase/ceftazidime-resistant, and only one isolate was quinolone-sensitive. Only one *S. aureus* culture was methicillin-sensitive.

The role of surgery in SBO is controversial. Surgery is considered a cornerstone of management by some and not so significant by others. In our cohort of patients, only two patients had complete debridement, one of whom died, and other patients who had surgery for obtaining samples for histopathology and cultures only had a reasonable outcome [2,6]. Hence, we conclude that surgery has a limited role in the management, and surgical interventions should mainly focus on obtaining a specimen for diagnostics and excluding malignancy [13,14].

Management of SBO is challenging and primarily based on culture-directed antimicrobials for an extended duration (three to six months) or until complete radiological and clinical recovery. Three to six months of culture-directed antimicrobial therapy is the preferred care, but successful treatment does not rely upon treatment duration alone. Other factors affecting the outcomes include the extent of involvement, sensitivity profile of pathogen isolated, polymicrobial versus monomicrobial growth, co-morbidities, and culture-negative SBO [15,16]. There are evolving records on using hyperbaric oxygen; however, its use is constrained because of unavailability [1-3,6,9].

Strengths and limitations

There is limited current data regarding craniofacial osteomyelitis, mostly in the form of small research studies or case reviews; hence, this study can offer further data for future research. Our data have many limitations as the study was retrospective. The study had a limited range of instances providing specialized care and might not constitute the actual occurrence within the society because our findings cannot be generalized to larger populations.

## Conclusions

SBO is a potentially fatal illness that poses diagnostic and treatment challenges. Clinical suspicion and good microbiological identification are critical components of a quick and accurate diagnosis. Prolonged intensive intravenous antibiotic therapy is required for treatment, which can be supplemented with surgical intervention in some circumstances. Paresis of the cranial nerves suggests illness progression and is associated with a lengthier hospital stay. A similar association is observed in patients requiring surgery for SBO. A history of diabetes can impede the healing process. For these individuals, prolonged outpatient parenteral antibiotic therapy provided via continuous infusion may be a beneficial choice. As more patients survive owing to advances in diagnostics and immune therapies, and as we treat more immunocompromised patients, infections are the most significant complications. Infections of the skull bones have become an established entity. We conclude that further large-scale studies and randomized controlled trials should be performed to set guidelines.
